# Efficacy and safety of IL-17 inhibitors for the treatment of ankylosing spondylitis: a systematic review and meta-analysis

**DOI:** 10.1186/s13075-020-02208-w

**Published:** 2020-05-12

**Authors:** Yufeng Yin, Mingjun Wang, Mengru Liu, Erye Zhou, Tian Ren, Xin Chang, Michun He, Keqin Zeng, Yufan Guo, Jian Wu

**Affiliations:** 1 Department of Rheumatology and Immunology, The First Affiliated Hospital of Soochow University, No.188 Shizi St, Suzhou, 215006 Jiangsu China; 2 Department of Rheumatology and Immunology, Ruijin Hospital, Shanghai Jiaotong University School of Medicine, Shanghai, China

**Keywords:** Ankylosing spondylitis, Interleukin inhibitors, Efficacy, Safety, Meta-analysis

## Abstract

**Objectives:**

To systematically assess the efficacy and safety of IL-17 inhibitors in patients with active ankylosing spondylitis.

**Methods:**

A systematic review of the literature was performed for randomized controlled trials (RCTs) concerning IL-17 inhibitors in patients with ankylosing spondylitis. Meta-analyses were used to determine the efficacy and safety of the IL-17 inhibitors in the treatment of these patients. The primary endpoint was predefined as the proportion of patients with at least 20% improvement in the Assessment of Spondyloarthritis International Society (ASAS20) response criteria at week 16, and the secondary endpoint was defined as ASAS40 at week 16.

**Results:**

Six phase III randomized, double-blind, placebo-controlled trials including 1733 patients (1153 patients received IL-17 inhibitors, including secukinumab or ixekizumab, whereas 580 patients received a placebo as comparators) were included. At week 16, the IL-17 inhibitor regimen produced a significant increase in the ASAS20 response rate (RR = 1.63, 95% CI 1.45 to 1.84, *p* = 0.00) and the secondary endpoint ASAS40 response rate (RR = 2.12, 95% CI 1.75 to 2.56, *p* = 0.00) versus those for the placebo. With respect to the safety profile, more treatment-emergent adverse events (RR = 1.11, 95% CI 1.01 to 1.22, *p* = 0.03) and non-severe infections (RR = 1.82, 95% CI 1.40 to 2.37, *p* < 0.001) were described after treatment with IL-17 inhibitors than after treatment with placebo, while no increased risk of other adverse events was indicated after IL-17 inhibitor therapy, including death, discontinuation due to adverse events, or serious adverse events.

**Conclusions:**

IL-17 inhibitors produced favorable response rates but an increased risk of non-severe infections in the treatment of active ankylosing spondylitis.

## Key messages

- Several IL-17 inhibitors such as secukinumab are approved for the treatment of ankylosing spondylitis.
- IL-17 inhibitors produce favorable response rates in patients with active ankylosing spondylitis.
- IL-17 inhibitors increase the risk of non-severe infections.

## Introduction

Ankylosing spondylitis is a chronic disease, with a prevalence ranging from 9 to 30 per 10,000 persons of the adult population worldwide [1, 2]. Ankylosing spondylitis is generally characterized by irreversible and structural damage of the sacroiliac and spinal joints, and finally, progressive spinal ankylosis due to new bone formation [3]. Clinical presentations of these patients include chronic back pain, morning stiffness, and loss of spinal mobility, which primarily affect the pelvis and the lower back [3]. For the treatment of active ankylosing spondylitis, non-steroidal anti-inflammatory drugs (NSAIDs) are still the first-line recommended treatment approach in the current primary management recommendations [4, 5]. On the other hand, there is no evidence that conventional synthetic disease-modifying antirheumatic drugs (csDMARDs), such as methotrexate and sulfasalazine, have any role in the treatment of axial manifestations, although these medications may slightly improve the peripheral symptoms that coexist with axial disease [6]. For patients who do not respond to or tolerate NSAIDs, biological disease-modifying antirheumatic drugs (bDMARDs), mainly tumor necrosis factor inhibitors (TNFis), have been recommended since the early 2000s [4, 5]. However, approximately 30 to 40% of patients have disease that remains active after standardized treatment with NSAIDs and/or bDMARDs [7–9]. The emergence of interleukin (IL)-17 inhibitors has changed the treatment landscape of active ankylosing spondylitis [10].

IL-17 is a pro-inflammatory cytokine that plays critical roles within a network of cytokines and is a key contributor to tissue repair on barrier surfaces, anti-infective immune responses, and the pathogenesis of various inflammatory diseases, especially ankylosing spondylitis [3]. This activity indicates that IL-17 blockade is a potential and efficient treatment option. During the past decade, there has been an increasing interest in the use of IL-17 inhibitors (such as secukinumab, ixekizumab, and brodalumab) in the treatment of psoriasis, psoriatic arthritis, and ankylosing spondylitis [11]. Secukinumab is a fully recombinant human immunoglobulin G (IgG)1 kappa monoclonal antibody (mAb) that directly inhibits IL-17A. It is the first approved IL-17 inhibitor for the treatment of patients with ankylosing spondylitis [12]. Increasing studies have displayed the efficacy of secukinumab in the treatment of ankylosing spondylitis after the first randomized controlled trials (RCTs) that focused on IL-17 blockade in ankylosing spondylitis [13]. Ixekizumab is another mAb specific for IL-17A that is approved for the treatment of moderate-to-severe plaque psoriasis and active psoriatic arthritis [14, 15]. Recently released results demonstrated that ixekizumab can also improve the clinical presentations of patients with ankylosing spondylitis [16, 17]. Brodalumab is a human mAb that binds with high affinity to human IL-17 receptor A (IL-17RA) and can lead to a significantly efficacious therapeutic alternative for psoriasis and psoriatic arthritis [18–20]. However, a clinical trial in psoriasis was aborted after several cases of suicide attempts, even though the association between IL-17RA inhibitor and suicidal tendency is still not confirmed [21]. Clinical trials focusing on several other IL-17 inhibitors, including bimekizumab and netakimab, are underway to evaluate their therapeutic potential in ankylosing spondylitis [10].

To date, a comprehensive overview of different IL-17 inhibitors as an entity in ankylosing spondylitis has not been satisfactorily explained. In the present study, we therefore assessed the efficacy and safety of IL-17 inhibitors in the treatment of ankylosing spondylitis via a systematic review and quantitative meta-analysis of data from the recently released RCTs.

## Methods

A systematic review and meta-analysis was accomplished following the recommendations of the Preferred Reporting Items for Systematic Reviews and Meta-Analyses (PRISMA) guidelines [22]. The authors declare that all supporting data are available within the article. This study did not require ethical approval or informed consent since all analyses were based on previously published data. The study protocol was prospectively registered on International Prospective Register of Systematic Reviews (PROSPERO) (registration ID: 157934).

### Data sources and search strategy

Bibliographic databases (including Medline via PubMed, EMBASE via OVID, and Web of Science) were searched for publications from their inceptions to 8 November 2019, without language restrictions. A combination of the following keywords was used: “axial spondyloarthritis,” “ankylosing spondylitis,” “anti-interleukin-17,” “anti-IL-17,” “IL17 receptor blockade,” “anti-IL17R,” “secukinumab,” “ixekizumab,” “brodalumab,” “bimekizumab,” and “netakimab.” The search was independently performed by two investigators (YY and ML), and discrepancies in the study selection were resolved by consensus. The search strategy is listed in online supplementary table S1. The references in the enrolled trials or meta-analyses were screened manually to find relevant original studies, and sponsored companies or study authors were contacted for additional information if ambiguity existed.

### Study selection

All relevant data from the RCTs comparing the effects and safety of IL-17 inhibitors with those of a placebo in ankylosing spondylitis were potentially eligible for inclusion. Studies fulfilling the following inclusion criteria were enrolled in the meta-analysis: (1) participants recruited in individual studies had active or radiographic ankylosing spondylitis and met the modified New York criteria [23], (2) IL-17 inhibitors (including secukinumab, ixekizumab, and brodalumab) were compared to a placebo in the treatment of these patients, and (3) efficacy of IL-17 inhibitors was measured using Assessment of Spondyloarthritis International Society response criteria for 20% improvement (ASAS20) and ASAS40 after a minimum duration of 16 weeks, and adverse events after receiving IL-17 inhibitors or the placebo were also reported. Exclusion criteria included a non-randomized design, a non-placebo comparative study, and an unqualified article type (abstracts without full-text publication, case reports, and duplications with the same samples). In order to minimize heterogeneity which might be caused by small sample size, studies that enrolled subjects less than 100 were also excluded.

### Data extraction and quality assessment

The full-text article of all studies that were potentially available for inclusion was read by two independent raters (YY and ML). Discrepancies at any stage of the data extraction process were resolved upon consensus. Data were collected based on a predefined template, including researcher names (authors), publication year, number of subjects, IL-17 inhibitor type, drug regimen, and efficacy endpoint. We also extracted data on adverse events, including any adverse event, death, discontinuation due to adverse event, infection, and serious adverse event. We assessed the quality of studies suitable for meta-analysis using the modified Jadad tool for randomized clinical trials [24]. Jadad contains two questions for randomization and masking and one question assessing the description of withdrawals and dropouts. Studies with no less than 3 points are ranked as high quality.

### Data synthesis and analysis

A meta-analysis was performed for three factors: efficacy evaluation of IL-17 inhibitors compared with that of a placebo by the primary outcome ASAS20 and secondary outcome ASAS40, IL-17 inhibitor efficacy assessment between TNFi-naïve patients and patients who had previous inadequate response or intolerance to an TNFi therapy (TNFi-IR), and a comparison of the safety profile between IL-17 inhibitors and the placebo. The meta-analyses were carried out using the Mantel-Haenszel method to determine the weight given to each study. A fixed-effects model was used when there was no significant heterogeneity, whereas a random-effects model was used in other cases. This produced a weighted estimate of the risk ratio (RR) with a 95% confidence interval (95% CI), considering the weight of the different samples. The heterogeneity amongst studies was examined on the basis of the *Q* test (*χ*^2^). The heterogeneity was qualified by the *I*^2^ statistic, ranging from 0 to 100%, in which high values of *I*^2^ represent strong heterogeneity. The sources of heterogeneity were explored by producing a Galbraith radial plot. The likelihood of publication bias was evaluated graphically by using sensitivity analysis. All statistical analyses were performed using RevMan statistical software version 5.3 (Nordic Cochrane Center, Copenhagen, Denmark) and Stata/MP version 13.0 (StataCorp, TX, USA). *p* values lower than 0.05 were considered significant.

## Results

### Literature search and study characteristics

**Fig. 1:**
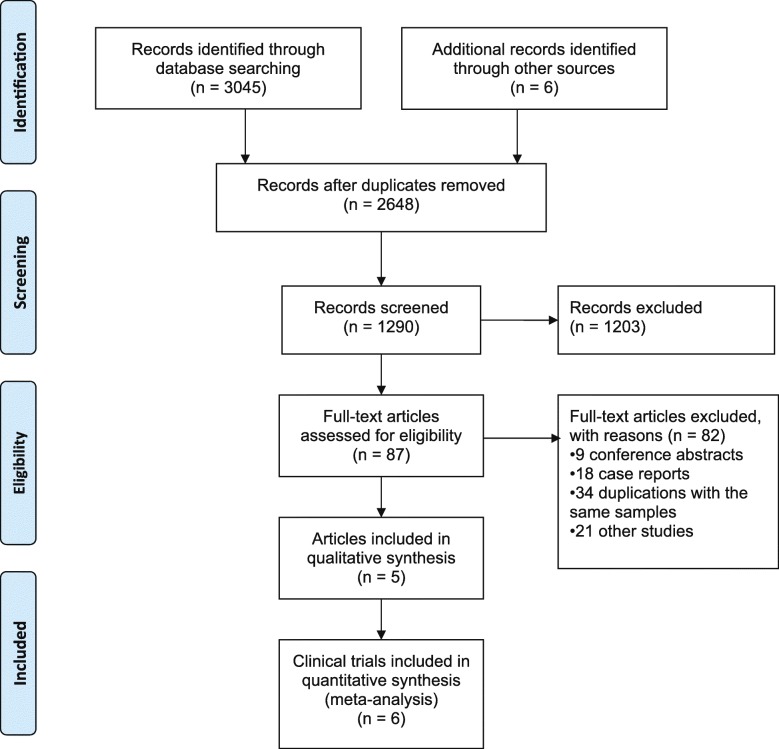
Flowchart of the search

Initially, 3051 potentially relevant citations were screened, and 2648 remained after duplicates were removed. The flowchart of the literature search is shown in Fig. 1. After manually searching the reference lists, our literature search finally identified five published articles including six clinical trials [25–29] with an overall 1733 patients (777 patients received secukinumab vs. 389 patients received a placebo, and 376 patients received ixekizumab vs. 191 patients received a placebo) that could be used in this meta-analysis. All studies were phase III randomized, double-blind, placebo-controlled trials. Secukinumab was evaluated in 4 trials of 3 published articles [25–27], and ixekizumab was used in two articles in the treatment of ankylosing spondylitis [28, 29]. No data concerning brodalumab therapy in ankylosing spondylitis were published through the date of literature retrieval. The ASAS20/40 response rate of treatment for ankylosing spondylitis at week 16 was reported in all six trials, while the ASAS partial remission rate was described in three trials [25, 26]. Similar large variations were observed for the proportion of male sex, ranging from 52% (MEASURE-3) to 83.7% (COAST-W), and the mean ± SD of age, ranging from 40.1 ± 11.6 years (MEASURE-1) to 47.4 ± 13.4 years (COAST-W). Patient characteristics are detailed in Table 1. The methodological qualities of all trials are high in light of the clear declaration of the randomization in patient selection, blinding, and outcomes of all patients in their trials.

**Table 1:** Main characteristics of the included studies

Trial (author)	Year	***N***	Treatment arms	Age (years)	Male (%)	Drug regimen	Endpoint
MEASURE-1 (Baeten) [25]	2015	125	SEC 150 mg	40.1 ± 11.6	84 (67)	i.v. 10 mg/kg at weeks 0, 2, and 4 and then 150 mg or 75 mg s.c. Q4W starting at week 8.	Primary: ASAS20 (week 16) Secondary: ASAS40 and ASAS partial remission (week 16)
		124	SEC 75 mg	42.3 ± 13.2	88 (71)		
		122	PBO	43.1 ± 12.4	85 (70)		
MEASURE-2 (Baeten) [25]	2015	72	SEC 150 mg	41.9 ± 12.5	46 (64)	s.c. 150 mg or 75 mg at weeks 0, 1, 2, 3, and 4 and then every 4 weeks.	Primary: ASAS20 (week 16) Secondary: ASAS40 and ASAS partial remission (week 16)
		73	SEC 75 mg	44.4 ± 13.1	51 (70)		
		74	PBO	43.6 ± 13.2	56 (76)		
MEASURE-3 (Pavelka) [26]	2017	76	SEC 300 mg	42.1 (11.8)	50 (65.8)	i.v. 10 mg/kg, weeks 0, 2, and 4; 300 mg or 150 mg or matched PBO, s.c. every 4 weeks.	Primary: ASAS20 (week 16) Secondary: ASAS40 and ASAS partial remission (week 16)
		74	SEC 150 mg	42.9 (11.1)	46 (62.2)		
		76	PBO	42.7 (11.4)	40 (52.6)		
MEASURE-4 (Kivitz) [27]	2018	116	Group 1*	44.5 ± 11.62	81 (69.8)	Group 1: s.c. 150 mg SEC load at weeks 0, 1, 2, and 3; group 2: s.c. 150 mg no load.	Primary: ASAS20 (week 16) Secondary: ASAS40 (week 16)
		117	Group 2^†^	41.2 ± 11.07	83 (70.9)		
		117	PBO	43.4 ± 12.46	76 (65.0)		
COAST-V (van der Heijde) [28]	2018	83	IXE Q2W	41.3 ± 11.2	68 (84)	s.c. 80 mg Q2W or Q4W.	Primary: ASAS40 (week 16) Secondary: ASAS20 (week 16)
		81	IXE Q4W	41.0 ± 12.1	64 (77)		
		87	PBO	42.7 ± 12.0	71 (83)		
COAST-W (Deodhar) [29]	2019	98	IXE Q2W	44.2 ± 10.8	75 (76.5)	s.c. loading dose (80 mg or 160 mg, respectively) and then 80 mg Q2W or Q4W thereafter.	Primary: ASAS40 (week 16) Secondary: ASAS20 (week 16)
		114	IXE Q4W	47.4 ± 13.4	91 (79.8)		
		104	PBO	46.6 ± 12.7	87 (83.7)		

*SEC* secukinumab, *IXE* ixekizumab, *PBO* placebo, *i.v.* intravenous injection, *s.c.* subcutaneous injection, *ASAS20/40* Assessment of Spondyloarthritis International Society response criteria for improvement of 20%/40%, *Q2W* every 2 weeks, *Q4W* every 4 weeks

*Secukinumab (150 mg) with a loading dose; ^†^secukinumab (150 mg) without a loading dose

### Overall treatment effect of IL-17 inhibitors

**Fig. 2:**
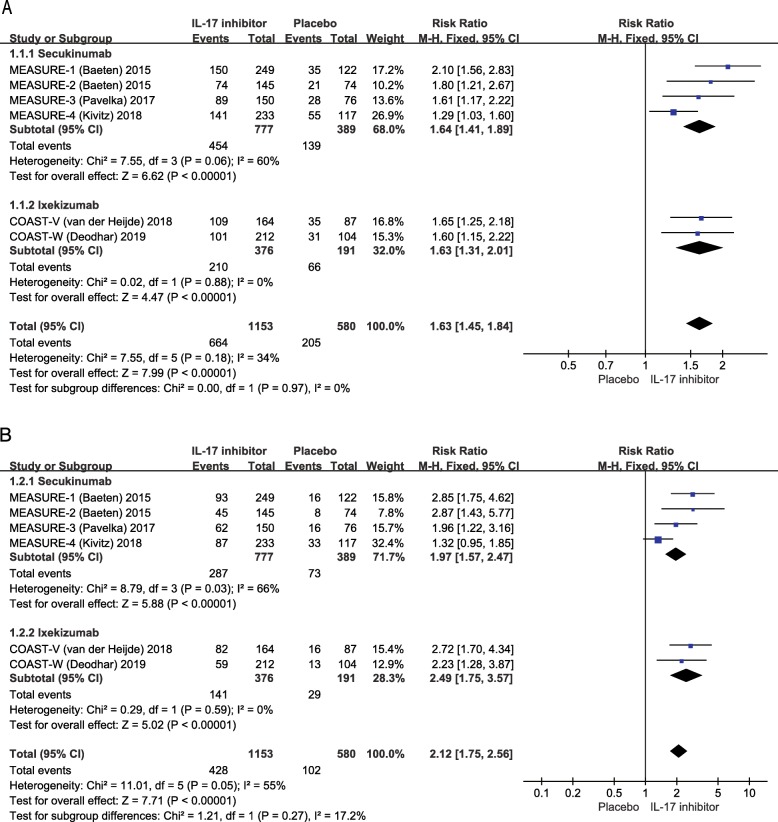
Forest plot of the efficacy of IL-17 inhibitors in the treatment of patients with ankylosing spondylitis, using ASAS20 (**a**) and ASAS40 (**b**). ASAS20/40, Assessment of Spondyloarthritis International Society response criteria for improvement of 20%/40%; RR, risk ratio

Amongst the six trials (four trials of secukinumab and two of ixekizumab) focusing on the efficacy of IL-17 inhibitors in ankylosing spondylitis, 1153 patients received IL-17 inhibitor therapy (777 of secukinumab and 376 of ixekizumab) and 580 patients received a placebo (389 patients were used as comparators for secukinumab and 191 for ixekizumab). Pooled analysis demonstrated that at week 16, the primary endpoint of the ASAS20 response rate was significantly increased in patients treated with any dosage and type of IL-17 inhibitor (57.6%, 664/1153) compared to placebo (35.3%, 205/580) (RR = 1.63, 95% CI 1.45 to 1.84, *p* < 0.001). Subgroup analysis suggested similar results for the comparison of both secukinumab (58.4%, 454/777) vs. placebo (35.7%, 139/389) (RR = 1.64, 95% CI 1.41 to 1.89, *p* < 0.001) and ixekizumab (55.9%, 210/376) vs. placebo (34.6%, 66/191) (RR = 1.63, 95% CI 1.31 to 2.01, *p* < 0.001). A *χ*^2^ test for heterogeneity did not indicate heterogeneity amongst the included trials in synthetic analysis (*I*^2^ = 34%, *p* = 0.18) and subgroup analysis for secukinumab (*I*^2^ = 60%, *p* = 0.06) and ixekizumab (*I*^2^ = 0%, *p* = 0.88) (Fig. 2a). The secondary endpoint of the ASAS40 response rate also had a significant increase in the IL-17 inhibitor regimen (37.1%, 428/1153) compared with that in the placebo treatment (17.6%, 102/580) (RR = 2.12, 95% CI 1.75 to 2.56, *p* < 0.001), and the subgroup analysis revealed an increased ASAS40 response rate with secukinumab (36.9%, 287/777) vs. placebo (18.8%, 73/389) (RR = 1.97, 95% CI 1.57 to 2.47, *p* < 0.001) and ixekizumab (37.5%, 141/376) vs. placebo (15.2%, 29/191) (RR = 2.49, 95% CI 1.75 to 3.57, *p* < 0.001). There was no heterogeneity across the included trials in pooled analysis (*I*^2^ = 55%, *p* = 0.05) and subgroup analysis of ixekizumab (*I*^2^ = 0%, *p* = 0.59), but an exception was found for secukinumab (*I*^2^ = 66%, *p* = 0.03) (Fig. 2b). Subgroup analysis based on the exposure to TNFis indicated a trend towards a higher ASAS20 response rate in TNFi-naïve patients (61.7%, 230/373) than in TNFi-IR patients (47.7%, 74/155) after IL-17 inhibitor therapy (RR = 1.27, 95% CI 1.06 to 1.52, *p* = 0.01), but no trend was observed in the ASAS40 response rate (40.2%,150/373 vs. 29.7%, 46/155) (RR = 1.31, 95% CI 1.00 to 1.72, *p* = 0.05) at week 16 (online supplementary figure S1).

### Heterogeneity analysis and publication bias

In the present study, since the *I*^2^ values of the efficacy analysis were more than 50%, as demonstrated above, a Galbraith radial plot was produced to explore the potential sources of heterogeneity. Our results suggested that no study was the major source of heterogeneity (online supplementary figure S2). A sensitivity analysis, by iteratively removing individual studies, was conducted to explore the impact of individual studies on the heterogeneity in this meta-analysis, and our findings suggested that there was no influence on the efficacy analysis after omitting any single-study estimates (online supplementary figure S3).

### Safety profile of IL-17 inhibitors

**Fig. 3:**
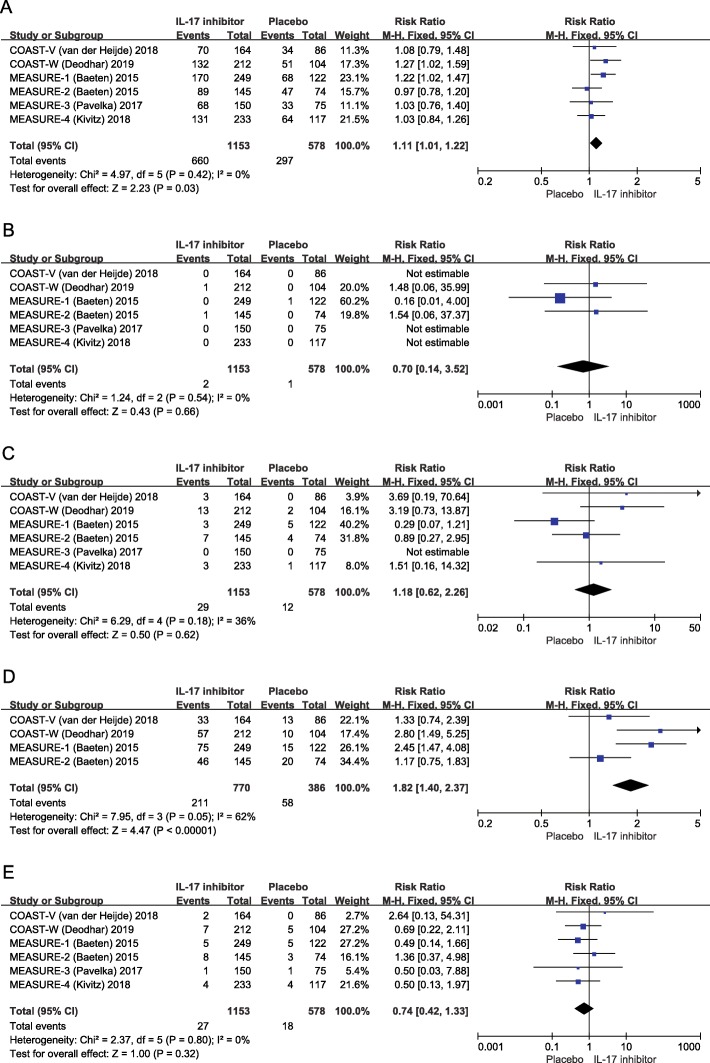
Forest plot of the safety profile of IL-17 inhibitors in the treatment of patients with ankylosing spondylitis in terms of treatment-emergent adverse events (**a**), death (**b**), discontinuation due to adverse event (**c**), non-severe infections (**d**), or serious adverse events (**e**). RR, risk ratio

The incidence of adverse events during the placebo-controlled periods was reported in all the included trials. Adverse events at week 16 were evaluated in this meta-analysis. After IL-17 inhibitor treatment, the most frequent adverse events reported were treatment-emergent adverse events (57.2%, 660/1153 vs. placebo 51.4%, 297/578) (RR = 1.11, 95% CI 1.01 to 1.22, *p* = 0.03) (Fig. 3a) and non-severe infections (27.4%, 211/770 vs. placebo 15.0%, 58/384) (RR = 1.82, 95% CI 1.40 to 2.37, *p* < 0.001) (Fig. 3d). The majority of infections were mild or moderate, with the most frequently reported being upper respiratory tract infections and nasopharyngitis. No discontinuation due to non-severe infections was reported in the included studies. There were no significant differences between IL-17 inhibitors and the placebo with regard to death (0.17%, 2/1153 vs. 0.17%, 1/578) (RR = 0.70, 95% CI 0.14 to 3.52, *p* = 0.86) (Fig. 3b), discontinuation due to adverse event (2.5%, 29/1153 vs. 2.1%, 12/578) (RR = 1.18, 95% CI 0.62 to 2.26, *p* = 0.62) (Fig. 3c), or serious adverse events (including reactivation tuberculosis, bacterial sepsis, or invasive fungal infections) (2.3%, 27/1153 vs. 3.1%, 18/578) (RR = 0.74, 95% CI 0.42 to 1.33, *p* = 0.32) (Fig. 3e). The safety profile of each trial is demonstrated in Table 2. A subsequent *Q* test showed that none of the single studies was homogeneous in this meta-analysis.

**Table 2:** Summary of safety outcomes at week 16

Trial (author)	Year	Treatment arms	Treatment-emergent adverse events, % (***n***/***N***)	Death, % (***n***/***N***)	Discontinuation due to adverse events, % (***n***/***N***)	Non-severe infections, % (***n***/***N***)	Serious adverse events, % (***n***/***N***)
MEASURE-1 (Baeten) [25]	2015	SEC	68.3 (170/249)	0 (0/249)	1.2 (3/249)	30.1 (75/249)	2.0 (5/249)
		PBO	55.7 (68/122)	0.8 (1/122)	4.1 (5/122)	12.3 (15/122)	4.1 (5/122)
MEASURE-2 (Baeten) [25]	2015	SEC	61.4 (89/145)	0.7 (1/145)	4.8 (7/145)	31.7 (46/145)	5.5 (8/145)
		PBO	63.5 (47/74)	0 (0/74)	5.4 (4/74)	27.0 (20/74)	4.1 (3/74)
MEASURE-3 (Pavelka) [26]	2017	SEC	45.3 (68/150)	0 (0/150)	0 (0/150)	N/A	0.7 (1/150)
		PBO	44 (33/75)	0 (0/75)	0 (0/75)	N/A	1.3 (1/75)
MEASURE-4 (Kivitz) [27]	2018	SEC	56.2 (131/233)	0 (0/233)	1.3 (3/233)	N/A	1.7 (4/233)
		PBO	54.7 (64/117)	0 (0/117)	0.9 (1/117)	N/A	3.4 (4/117)
COAST-V (van der Heijde) [28]	2018	IXE	42.7 (70/164)	0 (0/164)	1.8 (3/164)	20.1 (33/164)	1.2 (2/164)
		PBO	39.5 (34/86)	0 (0/86)	0 (0/86)	15.1 (13/86)	0 (0/86)
COAST-W (Deodhar) [29]	2019	IXE	62.3 (132/212)	0.5 (1/212)	6.1 (13/212)	26.9 (57/212)	3.3 (7/212)
		PBO	49.0 (51/104)	0 (0/104)	1.9 (2/104)	9.6 (10/104)	4.8 (5/104)

*SEC* secukinumab, *IXE* ixekizumab, *PBO* placebo, *N/A* not applicable

## Discussion

A systematic review and meta-analysis, being distinct from the individual studies, enables us to describe more comprehensive and accurate data by raising the persuasive power and resolution after synthesizing the outcomes of each analysis. To our knowledge, this study is the first meta-analysis focused on the efficacy and safety of IL-17 inhibitors over a placebo in patients with ankylosing spondylitis. Patients enrolled in these phase III clinical trials who received IL-17 inhibitor therapy showed, overall, significantly greater improvements in ASAS20/40 response rates than those not commencing equivalent treatment. Subgroup analysis after the division into secukinumab and ixekizumab confirmed these findings, even though the limited number of included trials requires a cautious interpretation of the findings. Concerning safety, pooled data across these clinical trials indicated that treatment with IL-17 inhibitors leads to a higher risk of curable non-fatal infection but not other severe adverse events, such as serious infection and infestation (including reactivation tuberculosis, bacterial sepsis, and invasive fungal infections) or death.

The main strength of the meta-analysis is that all the included studies were well-designed randomized, double-blind placebo-controlled trials. It provides a relatively large sample of patients with ankylosing spondylitis strictly recruited from more than 100 centers across Asia, Europe, and North and South America. Only mild heterogeneity was observed amongst the included studies. The findings of this meta-analysis of IL-17 inhibitors in ankylosing spondylitis agree with those meta-analyses examining the treatment of other rheumatic diseases. Previous meta-analyses have reported superior efficacy of secukinumab and ixekizumab over a placebo in achieving the American College of Rheumatology 20/50/70 risk ratios in the treatment of psoriatic arthritis [30, 31]. A more recent network meta-analysis using RCT data recruiting 12 types of bDMARDs in psoriatic arthritis found that both secukinumab and ixekizumab are safe and efficacious treatments for active psoriatic arthritis during induction therapy, and further analysis suggested a superiority of secukinumab to ixekizumab in the American College of Rheumatology criteria of ACR20 and a 75% improvement in psoriasis area and severity index (PASI75) [32]. Further systematic review suggested a significant resolution of dactylitis of IL-17 inhibitors in psoriatic arthritis at week 24 versus placebo [33]. Several other studies also suggested that IL-17 inhibitors, as a group, appeared to be effective and well tolerated in plaque psoriasis and rheumatoid arthritis [34–36]. Regarding the evaluation of IL-17 inhibitors in ankylosing spondylitis, one previous network meta-analysis compared the effectiveness of bDMARDs, including IL-17 inhibitors, for ankylosing spondylitis [37]. However, its results were unpersuasive on the evaluation of IL-17 inhibitors because only one early study [13] of secukinumab therapy with a relatively small sample size (24 participants receiving secukinumab and 6 placebo) was analyzed in this meta-analysis.

According to our pooled results, the use of IL-17 inhibitors does not increase the risk of adverse events, with the exception of mild infectious diseases, compared to that with a placebo. Two deaths were reported amongst all the included patients treated with IL-17 inhibitors. One death was caused by myocardial infarction in a patient subcutaneously receiving secukinumab (75 mg) in MEASURE-2, and one death due to suicide occurred after IL-17 inhibitor (ixekizumab) treatment in COAST-W [29]. This suicidal case was ultimately judged as unrelated to ixekizumab because the patient had a depression history of 1 year at study entry [29]. Apart from these cases, no suicidal ideation was reported across the included trials. There was no difference in the incidence of severe adverse events between IL-17 inhibitors (ranging from 0.7 to 5.5%) and the placebo (0–4.8%) in either the pooled results of our meta-analysis or the individual studies. The overall infection rate was not reported in MEASURE-3 and MEASURE-4, and the number of infectious diseases (including those of the ear, upper respiratory tract, and urinary tract infection and *Candida* infection) in patients treated with IL-17 inhibitors seemed to be more than that in those treated with a placebo, although statistical significance was not reached in these two trials. Finally, these adverse events did not induce an increased incidence of discontinuation of patients. These findings were consistent with those from studies of IL-17 inhibitors in psoriasis, psoriatic arthritis, and rheumatoid arthritis [30–32, 34, 36].

The current study has several unavoidable limitations. First, the correlation with treatment response rate for IL-17 inhibitors in ankylosing spondylitis might be dosage-dependent. Secukinumab (150 mg or 300 mg) and ixekizumab (80 mg Q1W or Q4W) induced similar improvements in the ASAS20/40 response rate [26–29], while the lower dosage of secukinumab (75 mg) resulted in significant improvements in signs and symptoms only with a higher intravenous loading dose [25]. Due to the limitation of the data, however, a subgroup analysis based on different dosages was unable to be conducted. These limitations might impair the power of our findings. Second, the short-term ASAS20 response rate achieved after secukinumab treatment was, for example, numerically different in TNFi-naïve subjects versus TNFi-IR subjects [25]. Our meta-analysis by TNFi experience also demonstrated that the overall ASAS20 response rate was statistically higher in TNFi-naïve patients than in TNFi-IR patients at week 16. In the single studies, the long-term (2-year) response rates were similar between TNFi-naïve subjects (observed ASAS20/40 data were 76.1 to 86.6%/56.3 to 65.7%) versus TNFi-IR subjects (observed ASAS20/40 data were 72.7 to 85.0%/40.9 to 68.8%, respectively) [27, 38]. The literature concerning the difference in the efficacy of IL-17 inhibitors according to history of TNFi treatment still remains unconfirmed. These findings should, therefore, be interpreted cautiously due to the limitation of the disclosure data.

## Conclusions

The findings of this meta-analysis suggest that IL-17 inhibitors are significantly effective in improving ASAS20/40 response rates at week 16 in patients with active ankylosing spondylitis. IL-17 inhibitors seem to be associated with higher ASAS20 response rates in TNFi-naïve subjects than TNFi-IR subjects. Meanwhile, there is a higher incidence of infectious diseases attributable to IL-17 inhibitors than to placebo, with most infections being mild or moderate. IL-17 inhibitors can be considered a favorable option for patients with active ankylosing spondylitis, especially TNFi-IR subjects.

## Supplementary information

**Additional file 1: Supplementary table S1.** Search strategy (Medicine via PubMed, EMBASE via OVID, and Web of Science). **Supplementary figure S1.** Forest plot of efficacy of IL-17 inhibitors in TNFi-naïve patients versus TNFi-IR patients in treatment of patients with ankylosing spondylitis using ASAS20 (A) and ASAS40 (B). ASAS20/40, Assessment of Spondyloarthritis International Society response criteria for improvement of 20%/40%. RR, risk ratio. TNF, tumor necrosis factor; IR, inadequate response. **Supplementary figure S2.** Galbraith radial plot for the assessment of heterogeneity sources in the analysis of ASAS20 (A) and ASAS40 (B). Each circle represents an individual study with larger circles representing larger sample sizes. No study was shown to be the source of the heterogeneity. ASAS20/40, Assessment of Spondyloarthritis International Society response criteria for improvement of 20%/40%. **Supplementary figure S3.** Sensitivity analysis for detecting publication bias in the analysis of ASAS20 (A) and ASAS40 (B). ASAS20/40, Assessment of Spondyloarthritis International Society response criteria for improvement of 20%/40%.

## Data Availability

All data generated and analyzed during this study are included in this published article.

## Abbreviations

ACR: American College of Rheumatology
ASAS: Assessment of Spondyloarthritis International Society
bDMARD: Biologic disease-modifying antirheumatic drug
csDMARD: Conventional synthetic disease-modifying antirheumatic drug
DMARD: Disease-modifying antirheumatic drug
NSAID: Non-steroidal anti-inflammatory drug
PRISMA: Preferred Reporting Items for Systematic Reviews and Meta-Analyses
PROSPERO: Prospective Register of Systematic Reviews
RCT: Randomized controlled trial
RR: Risk ratio
SpA: Spondyloarthritis
TNFi: Tumor necrosis factor inhibitor
95% CI: 95% confidence interval
