# Supplementary material for: Efficacy and safety of IL-17 inhibitors for the treatment of ankylosing spondylitis: a systematic review and meta-analysis
Source: Arthritis Res Ther. 2020 May 12;22:111. doi: 10.1186/s13075-020-02208-w (PMC7216398; doi:10.1186/s13075-020-02208-w)
Supplement: Supplementary file 1 — Additional file 1: Supplementary table S1. Search strategy (Medicine via PubMed, EMBASE via OVID, and Web of Science). Supplementary figure S1. Forest plot of efficacy of IL-17 inhibitors in TNFi-naïve patients versus TNFi-IR patients in treatment of patients with ankylosing spondylitis using ASAS20 (A) and ASAS40 (B). ASAS20/40, Assessment of Spondyloarthritis International Society response criteria for improvement of 20%/40%. RR, risk ratio. TNF, tumor necrosis factor; IR, inadequate response. Supplementary figure S2. Galbraith radial plot for the assessment of heterogeneity sources in the analysis of ASAS20 (A) and ASAS40 (B). Each circle represents an individual study with larger circles representing larger sample sizes. No study was shown to be the source of the heterogeneity. ASAS20/40, Assessment of Spondyloarthritis International Society response criteria for improvement of 20%/40%. Supplementary figure S3. Sensitivity analysis for detecting publication bias in the analysis of ASAS20 (A) and ASAS40 (B). ASAS20/40, Assessment of Spondyloarthritis International Society response criteria for improvement of 20%/40%. [file 13075_2020_2208_MOESM1_ESM.docx]

### Supplementary table S1. Search strategy (Medicine via PubMed, EMBASE via OVID, and Web of Science).

| **Database** | **Search strategy** |
| --- | --- |
| PubMed | (((psoriatic arthritis) OR ankylosing spondylitis)) AND (((((((anti‐interleukin‐17) OR anti‐IL‐17) OR IL17 receptor blockade) OR anti‐IL17R) OR brodalumab) OR ixekizumab) OR secukinumab)  **Searching result: N=383 (November 16, 2019)** |
| Embase | #1 'psoriatic arthritis'/exp OR 'ankylosing spondylitis'/exp  #2 anti‐interleukin‐17 OR anti‐il‐17 OR (il17 AND receptor AND blockade) OR anti‐il17r OR 'brodalumab' OR brodalumab OR ixekizumab OR secukinumab  #3 #1 AND #2  **Searching result: N=1,616 (November 16, 2019)** |
| Web of Science | #1 TOPIC: (psoriatic arthritis) OR TOPIC: (ankylosing spondylitis)  Indexes=SCI-EXPANDED, SSCI, A&HCI, CPCI-S, CPCI-SSH, BKCI-S, BKCI-SSH, ESCI, CCR-EXPANDED, IC Timespan=All years  #2 TOPIC: (anti‐interleukin‐17) OR TOPIC: (anti‐IL‐17) OR TOPIC: (IL17 receptor blockade) OR TOPIC: (anti‐IL17R) OR TOPIC: (brodalumab) OR TOPIC: (ixekizumab) OR TOPIC: (secukinumab)  Indexes=SCI-EXPANDED, SSCI, A&HCI, CPCI-S, CPCI-SSH, BKCI-S, BKCI-SSH, ESCI, CCR-EXPANDED, IC Timespan=All years  #3 #1 and #2  **Searching result: N= 1,046 (November 16, 2019)** |

### Supplementary figure S1. Forest plot of efficacy of IL-17 inhibitors in TNFi-naïve patients versus TNFi-IR patients in treatment of patients with ankylosing spondylitis using ASAS20 (A) and ASAS40 (B). ASAS20/40, Assessment of Spondyloarthritis International Society response criteria for improvement of 20%/40%. RR, risk ratio. TNF, tumor necrosis factor; IR, inadequate response.

**A**


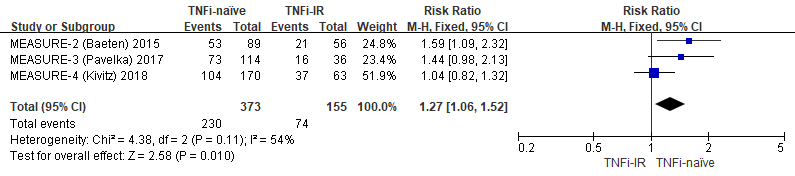


**B**


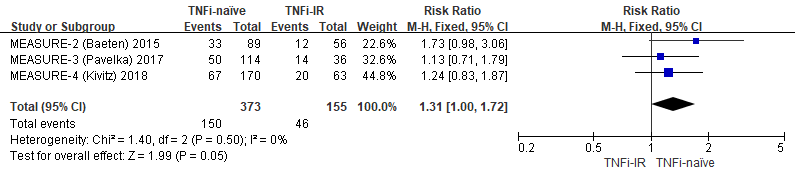


### Supplementary figure S2. Galbraith radial plot for the assessment of heterogeneity sources in the analysis of ASAS20 (A) and ASAS40 (B). Each circle represents an individual study with larger circles representing larger sample sizes. No study was shown to be the source of the heterogeneity. ASAS20/40, Assessment of Spondyloarthritis International Society response criteria for improvement of 20%/40%.

**A B**

### Supplementary figure S3. Sensitivity analysis for detecting publication bias in the analysis of ASAS20 (A) and ASAS40 (B). ASAS20/40, Assessment of Spondyloarthritis International Society response criteria for improvement of 20%/40%.

**A B**
